# Genome-wide DNA methylation analysis in obsessive-compulsive disorder patients

**DOI:** 10.1038/srep31333

**Published:** 2016-08-16

**Authors:** Weihua Yue, Weiqiu Cheng, Zhaorui Liu, Yi Tang, Tianlan Lu, Dai Zhang, Muni Tang, Yueqin Huang

**Affiliations:** 1 Peking University Sixth Hospital & Institute of Mental Health, Beijing 100191, China; 2 National Clinical Research Center for Mental Disorders & Key Laboratory of Mental Health, Ministry of Health (Peking University), Beijing 100191, China; 3 Department of Mental Health, Guangdong Provincial People’s Hospital, Guangzhou, China; 4 Peking-Tsinghua Center for Life Sciences/PKU-IDG/McGovern Institute for Brain Research, Peking University, Beijing 100871, China; 5 Guangzhou Psychiatry Hospital, Guangzhou, 510080, China

## Abstract

Literatures have suggested that not only genetic but also environmental factors, interactively accounted for susceptibility of obsessive-compulsive disorder (OCD). DNA methylation may regulate expression of genes as the heritable epigenetic modification. The examination for genome-wide DNA methylation was performed on blood samples from 65 patients with OCD, as well as 96 healthy control subjects. The DNA methylation was examined at over 485,000 CpG sites using the Illumina Infinium Human Methylation450 BeadChip. As a result, 8,417 probes corresponding to 2,190 unique genes were found to be differentially methylated between OCD and healthy control subjects. Of those genes, 4,013 loci were located in CpG islands and 2,478 were in promoter regions. These included *BCYRN1, BCOR, FGF13, HLA-DRB1, ARX*, etc., which have previously been reported to be associated with OCD. Pathway analyses indicated that regulation of actin cytoskeleton, cell adhesion molecules (CAMs), actin binding, transcription regulator activity, and other pathways might be further associated with risk of OCD. Unsupervised clustering analysis of the top 3,000 most variable probes revealed two distinct groups with significantly more people with OCD in cluster one compared with controls (67.74% of cases *v.s.* 27.13% of controls, Chi-square = 26.011, *df* = 1, *P* = 3.41E-07). These results strongly suggested that differential DNA methylation might play an important role in etiology of OCD.

Despite the obsessive-compulsive disorder (OCD), as a chronic neuropsychiatric disorder, affects 2% of the world population^1^,^2^, up to now, there has been no exact biomarkers to aid the clinician identifying this disorder. It has been reported that significant interactions with social, genetic, and work functioning might play important roles in the pathogenesis of OCD. Family studies showed that the OCD risk four to ten-fold risk increased among the patients’ first-degree relatives, when compared with healthy controls’. Linkage studies have identified chromosomes 3q, 9p, 10p, 15q, 4q, and 19q as linkage regions for OCD. The association studies have also reported that *DLGAP1, BTBD3, SLC64A, 5HT2A, DRD4*, etc., as appealing susceptibility genes for OCD. On the other side, environmental risk factors, such as vitamin B12, flotate, homocysteine and others, have also been reported to play important roles in the pathogenesis of OCD^3^. Moreover, childhood trauma, especially emotional abuse and neglect, may also increase the risk of mood disorders^4^.

Although individuals’ genome structure has been inherently fixed throughout lifetime, the epigenetic mechanism has been verified to be implicated in the regulation of gene expression. Hillemacher *et al*. reported that there were significant hypermethylation of dopamine transporter (*DAT*) promoter in subjects with alcohol dependence who were evaluated for obsessive-compulsive drinking^5^. Therefore, we put forward a hypothesis that abnormal methylation may play an important role in the pathogenesis of OCD. However, to our knowledge, no genome-wide methylation analysis of OCD has been reported up to now.

In the present study, we implemented a genome-wide DNA methylation study of OCD in Chinese Han population. According to differentially methylated loci, results of future epigenetic exploration may hold great promises of biomarkers and treatment for OCD, considering epigenetic processes might be reversed.

## Results

### Differential methylation

Sixty-five patients with OCD and 96 healthy control subjects showed no significant differences in terms of their demographic data, such as age and gender (Table 1).

**Figure 1:**
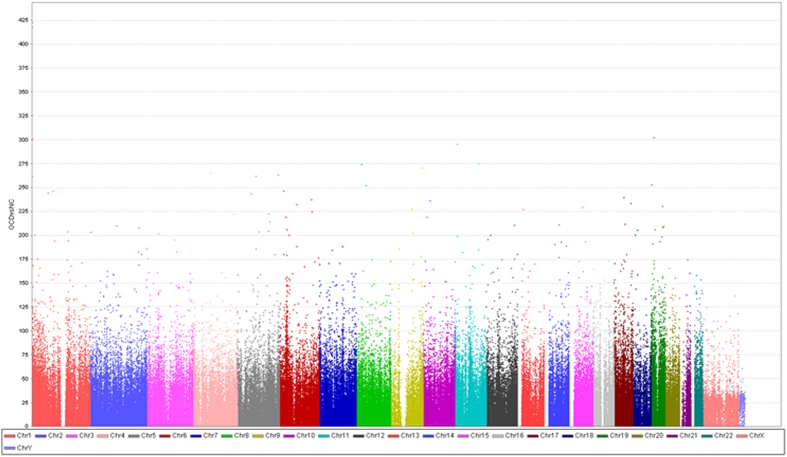
Manhantton plot across genome wide methylation. Chromosomal distribution of DNA methylation read of Illumina IHumanMethylation450 probes. X-axis represents position of probes across 22 autosomal chromosomes. Y-axis is the read count mapped in each chromosome.

**Figure 2:**
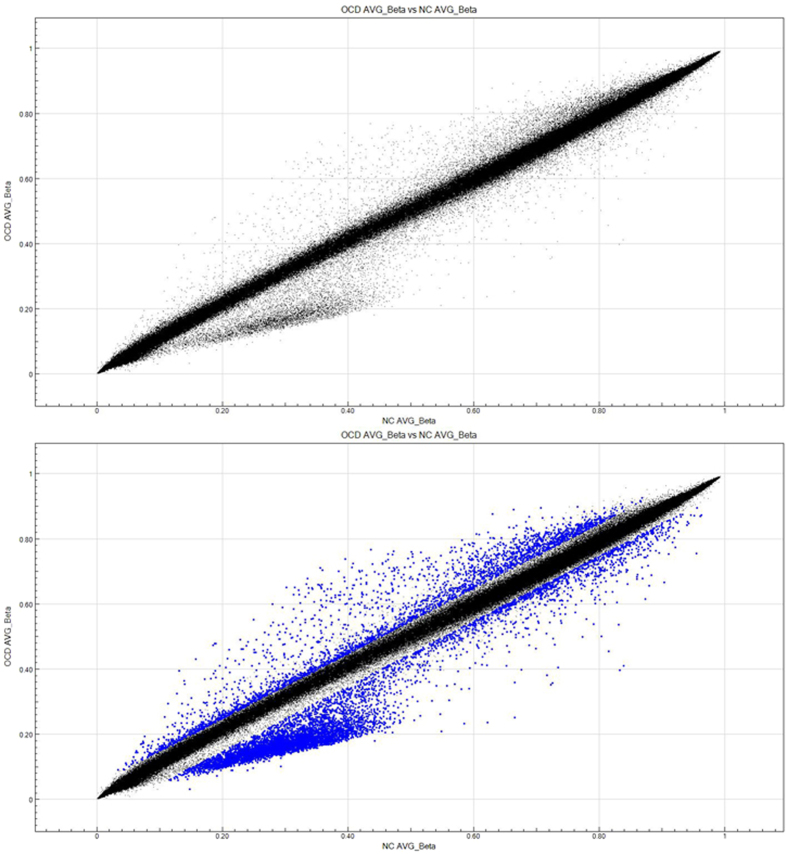
Scatter plots of probes intensity measuring the methylation level. X- and Y-axis represented the average *β-* values in control subjects and that in OCD patients, respectively. The blue dots are the significant probes which met the following two criteria: diffscore ≥20 (*P* < 0.01), as well as delta *β-*value ≥ 0.05.

Between two groups, a total of 8,417 probes were identified as differentially methylated (Fig. 1 and Table 2). Approximately 72.62% of probes (n = 6,116) were differentially methylated in OCD group, compared with healthy control subjects. The 8,417 probes corresponded to 2,190 unique genes. Among those significant probes, 2,478 loci were in a promoter-associated region of 388 genes (surrounding the transcription start site), 4,013 loci located in CpG islands of 937 genes. Among of those loci, 1,622 were both promoter-associated and located at CpG islands (Table 2). Moreover, the multidimensional scaling plots on adjusted *β-* values further reinforced the distinction of probes intensity for methylation level between OCD patients and healthy control subjects (Fig. 2).

As expected, altered DNA methylation mostly occurred in the CpG islands (28.39% unadjusted, 33.12% adjusted; 4,013/8,417). The proportion of probes in a CpG islands found differentially methylated in the adjusted results is significantly higher than overall proportion of probes found in CpG islands on the array as a whole (overall 31.13%; *P* = 1.26E-05). Furthermore, a high percentage of aberrant DNA methylation occurred in promoter- associated regions (12.83% unadjusted, 17.82% adjusted; 2,478/8,417). The proportion in the unadjusted results was significantly smaller than the proportion of promoter regions found on the arrays as a whole (overall 21.34%; *P* = 6.08E-04).

A gene list was generated based on genes previously found to be associated with OCD from genetic and/or DNA methylation studies, which included brain cytoplasmic RNA 1 (*BCYRN1*), BRO-c (*BCOR*), fibroblast growth factor 13 (*FGF13*), aristaless related homeobox (*ARX*), ephrin B1 (*EFNB1*); inhibitor of kappa light polypeptide gene enhancer in B-cells, kinase gamma (*IKBKG*); translocase of inner mitochondrial membrane 17 homolog B (yeast) (*TIMM17B*); polyglutamine binding protein 1 (*PQBP1*); glucose-6-phosphate dehydrogenase (*G6PD*); Rho GTPase activating protein 6 (*ARHGAP6*); chloride voltage-gated channel 5 (*CLCN5*), and so on. These genes were identified as differentially methylated after adjustment (Table 3). In the present study, some other genes with more than 3 significantly differential methylated loci including 5-hydroxytryptamine (serotonin) receptor 2C (*5HT2C*), dopamine receptor D5 (*DRD5*), BTB (POZ) domain containing 3 (*BTBD3*), which have also been previously reported to be associated with risk of OCD^3^,^4^,^5^,^6^. Stewart *et al*.^7^ reported one SNP rs6131295 near *BTBD3* gene associated with OCD at genome-wide significance (*P* = 3.84E-08). Furthermore, rs6131295 showed a cis-eQTL effect on BTBD3 in the frontal cortex (*P* = 0.028), a region that has repeatedly been implicated in OCD. In the present study, we found that *BTBD3* gene also showed hypomehtylation in OCD patients when compared with healthy control subjects. Our findings, as well as, previously published susceptible genes of OCD, together suggested there might be much more genetic or epigenetic abnormalities in OCD.

### Cluster analysis

We made clustering analysis for the top significant probes of differential methylation, which showed significantly methylation with *diffscore* ≥ 20, delta *β* > 0.1, *P* < 0.01. Adjusting the age and gender, *β-*values revealed two distinct clusters, one containing mainly OCD patients (67.74%), the other containing mainly controls (72.87%; Supplementary Figure 2). Cluster 1 had significantly more patients with OCD compared with healthy control subjects (Chi-square = 26.011; *df* = 1; *P* = 3.41E-07). Interpreting the results from these three analyses in conjunction, we confirmed that at least 12 out of the 65 patients are clearly distinguishable from controls.

For pathway analyses, we found several pathways involving differentially methylated genes associated with OCD, such as regulation of actin cytoskeleton (KEGG hsa04810), cell adhesion molecules (CAMs, KEGG hsa04514), actin binding (GO:0003779), transcription regulator activity (GO: 00016563), etc. (Table 4).

## Discussion

In the present study, we reported the differential DNA methylation in OCD, employing a genome-wide methylation array with very extensive coverage of the potential methylation sites in the human genome. To our knowledge, this is the first report about genome wide association of differentially methylated gene of OCD between those with OCD and control subjects in Chinese Han population. After adjusting for age, 8,417 probes corresponding to 2,190 unique genes were found to be differentially methylated. When comparing differentially methylated gene list with past studies using peripheral blood samples, we found a high concordance rate, particularly for genes previously found to be associated with OCD.

One of the genes we identified was the brain cytoplasmic RNA 1 (*BCYRN1*), BCL-6 co-repressor (*BCOR*), which has altered DNA methylation in the present study as well as other methylation studies. *BCYRN1* encodes a neural small non-messenger RNA, is a member of the family of interspersed repetitive DNA, which has been reported to play a role in Alzheimer’s diseases and cancers^8^. BCOR has been reported to regulate mesenchymal stem cell function by epigenetic mechanisms^9^. Other genes found to be differentially methylated in present the peripheral leukocyte tissue study includes *FGF13, ARX, HLA-DRB1,* etc., which have also been reported to participant in other disease associated with epigenetics^10^,^11^,^12^,^13^. Moreover, functional significance of genes found to be differentially methylated should also be tested by gene expression.

In the present study, we used the genome-wide peripheral blood leukocyte DNA methylation microarrays, as well as unsupervised clustering of the top significant probes of differential methylation revealed two distinct groups after adjusting for age gender, and methylation PCs. Cluster 1 comprised 67.74% patients with OCD and 27.13% controls, whereas cluster 2 comprised 32.26% patients with OCD and 72.87% controls.

Clustering analysis also revealed two subgroups within OCD. Maybe these two subgroups have specific symptom profiles need further investigation in samples with a comprehensive clinical history. For OCD, several literatures reported that genetic and environmental risk factors might contribute to the susceptibility of the disease. However, there have seldom literatures reported specific methylation abnormalities contributing to the risk or the disease. After adjusting for potential confounding factors (age, gender, methylation PCs, sampling points 1 for Beijing and 2 for Guangzhou), *BROC, BCYRN1, HLA-DRB1, ARX*, etc., were found to be differentially methylated between OCD patients and healthy control subjects. Interestingly, these were genes that we have previously found to be associated with OCD. Ciesielski *et al*. reported that a locus near the transcription start site of the leptin receptor (cg21655790) showed decreased methylation levels in infants of mothers with prenatal psychiatric disease (depression, anxiety, OCD or panic)^6^. Moreover, Stewart *et al*. reported the first genome-wide association study (GWAS) for OCD with multiethnic samples, which including the *DLGAP1, BTBD3* associated with OCD in (*P* < 10^−6^)^7^. In the present study, we also found 3 differential methylated sites on *BTBD3* and 2 sites on *DLGAP2*, which suggested the two genes might play an important roles in the pathogenesis of OCD. However, the potential implications should be further explored in the future.

Sabunciyan *et al*., also reported the first genome-wide DNA methylation scan in major depression disorder (MDD), and they found the greatest DNA methylation differences were in PRIMA1, with 12–15% increased DNA methylation in MDD (*P* = 0.0002–0.0003)^14^. In the present study, we found multiple genes showed methylation abnormalities in OCD. Türksoy reported that the importance of vitamin B12, folate, and homocysteine in carbon transfer metabolism (methylation), which was required for the production of serotonin as well as for other monoamine neurotransmitters and catecholamines^3^.

Our data indicate that studies of epigenetic changes in OCD hold promise for the future development of diagnostic and prognostic biomarkers for OCD, as well as therapeutic options that target causative epigenetic alterations. We wish to identify aberrant DNA methylation profiles in blood tissues and to determine whether the findings could be translated back into a diagnostically feasible tissues such as blood or saliva. Identifying when DNA methylation changes occur will be also important in understanding the origins of OCD. Literatures suggested that altered DNA methylation may occur during the critical periods of development between pregnancy and birth^15^. If gene-environmental risk factors affecting DNA methylation status could be identified, the incidence of OCD could possibly be reduced by targeting the environmental triggers. Putting together all the pieces of the puzzle of pathogenesis of OCD, it should to be further explored how the gene- environment interactions influence epigenetic progress. Over these years, there have been numerous studies on the effects of genetic polymorphisms on mRNA expression in OCD, but few researches indicating how polymorphisms affect gene expression through DNA methylation. Investigating effects of polymorphisms- environment interactions on epigenetics, will further benefit our understanding of the pathophysiology of OCD. To identify enzymes mediating DNA demethylation in cell lines as potential targets for therapeutic intervention also will be an exciting prospect, which may hold the key to reverse the psychiatric illness^16^.

The present study is a very preliminary exploration of methylation in OCD, partly because of a very small sample size and enrolling subjects from separate two hospitals, which may produce selection bias. On the other hand, we have over excluded some cases with other physical diseases, which may somewhat limited the sample size. In the future, we will enlarge the sample size and also verify our preliminary findings in an independent sample. Another limitation of the present study was that patients were not free of anti-OCD medication, and antipsychotic medication might influence DNA methylation^17^. In the future, we’ll recruit the drug-naïve patients with OCD, using much appropriate criteria, and will explore the relationship between methylation and therapeutic effects of anti-OCD medications.

## Methods

The methods were carried out in accordance with the approved guidelines.

### Ethics statement

This study was approved by the Medical Research Ethics Committee of the Institute of Mental Health, the Sixth Hospital, Peking University, and Guangzhou Psychiatry Hospital. Written informed consent was obtained from all participants.

### Subjects

Sixty-five OCD patients and 96 healthy control subjects were enrolled from the Sixth Hospital, Peking University and Guangzhou Psychiatry Hospital, respectively. The consensus diagnoses were made by at least two experienced psychiatrists, according to the criteria for primary OCD from the Diagnostic and Statistical Manual of Mental Health (DSM-IV)^18^. All of the cases, controls or their parents born in northern regions of China. Exclusion criteria included either severe chronic physical diseases, such as cancer, diabetes, asthma, cancer and heart diseases, epilepsy, or other primary psychiatric disorder, according to the DSM-IV criteria, comorbid diagnosis of schizophrenia, bipolar disorder, alcohol abuse, and other neurological disorders.

The severity of OCD was assessed by the Yale-Brown Obsessive Compulsive Scale (Y-BOCS)^19^. The mean age at onset of OCD was 22.1 ± 8.5 years and the mean duration of OCD was 32.4 ± 10.1 months in the case group. The healthy control subjects and related data came from the database of Key Laboratory of Mental Health of Ministry of Health (Sixth Hospital), Peking University. They are all northern Chinese. To control the basic element of case-control design, we just enrolled the control matched to each case by age (±5 years) and sex for further analysis. The healthy control subjects were also excluded if they met diagnosis or with history of OCD or other psychiatric disorders according to DSM-IV or physical diseases. The frequency matching methods for age and sex were used between 65 OCD cases and 96 healthy control groups (*p* > 0.05, Table 1).

### Illumina Infinium HumanMethylation450 Beadchip

Genomic DNA samples were extracted using the QIAamp DNA Mini Kit (QIAGEN) and stored at −80 °C. Quality checking of the samples was performed by Nanodrop Spectrophotometer (Nanodrop, Wilmington, DE, USA) and resolution on a 0.8% agarose gel. Samples were bisulphite converted with Zymo EZ DNA Methylation kit (Zymo Research, Irvine, CA, USA). GenomeStudio ChIP Sequencing Module v2011.1 (Illumina, San Diego, CA, USA) with Methylation module 1.9.0 software with the default Illumina settings and Illumina HumanMethylation450 manifest files was used in the methylation analysis^20^,^21^. The Infinium platform assays include more than 485,000 CpG sites, encompassing 99% of RefSeq genes. The Beadchip covers 96% of CpG islands, shores (within 2 kb from CpG islands) and shelves (>2 kb from CpG islands), as well as. It can also cover sites outside of CpG islands, DNase hypersensitive sites, as well as the incorporating miRNA promoter regions. All the Illumina quality controls included the following controls: sample-independent, sample-dependent, staining, extension, target removal, hybridization, bisulphite conversion I and II, specificity, non-polymorphic, and negative controls^22^.

### Data processing

The Illumina GenomestudioV2011 program (Illumina Ltd.) was used to analyze BeadArray data to assign site-specific DNA methylation *β-*values to each CpG site. The Beadchips were subjected to fluorescently labeled nucleotides of two different colors, each corresponding to the cytosine (methylated, M) or uracil (unmethylated, U) identity of the bisulfite- converted nucleotide at a specific CpG site. The proportion of methylation (*β*) were calculated as: *β* = Max(M, 0)/[Max(M, 0) + Max(U, 0) + 100]. The methylation status for each probe was calculated as a *β*- value (ranged 0~1), which representing low to high levels of methylation^21^,^22^. After further filtering of overlaps, 386,135 probes were leaving for final analysis. *P* > 0.05 indicated that the data points were not significantly different from background measurements.

### Differential methylation detection

In order to assess differences in methylation between case and control groups, the original 386,135 *β*-values were converted to *M-*values via the logit transformation^23^. Differentially methylated probes were detected using the Limma package of Bioconductor (version 3.2)^23^,^24^,^25^. Used the one-sample Kolmogorov-Smirnov test for exploring the distribution test of *β*-value of methylation, we found the *z*-value of *K-S* test was 0.493, which suggested that the distribution was normal (Gaussian distribution). Therefore, we used the linear regression analysis for further detecting differentially methylated probes of the *β*-value between two groups, with adjusted potential confounding covariate factors (age, gender, methylation PCs, sampling points 1 for Beijing and 2 for Guangzhou). The Bonferroni, Benjamini-Hochberg and false discovery rate (FDR) methods were used to adjust the *P*-values^26^. Probes were considered to be differentially methylated if adjusted *P* < 0.05. The corresponding genes were annotated derived from the gene annotations associated with the probes.

### Unsupervised clustering analysis

We selected top 3,000 most significant different methylated probes for further cluster analyses, using the recursively partitioned mixture model (RPMM)^27^,^28^. To adjust for age, a linear model was fitted to the *M-* values (logit- transformed *β-*values) with the Limma package and the R software. To visualize the distance between samples and to further reinforce the clustering, multidimensional scaling with a Euclidian distance was performed (Supplementary Figure 2).

### Pathway analysis

We selected the differentially methylated genes with (Freq ≥ 2) for functional and pathway-based annotation. The pathway data were extracted from two typical and public pathway databases, Kyoto Encyclopedia of Genes and Genomes (KEGG) (http://www.kegg.jp/kegg/pathway.html), as well as Gene Ontology (GO) (www.geneontology.org) (Table 4).

## Additional Information

**How to cite this article**: Yue, W. *et al*. Genome-wide DNA methylation analysis in obsessive-compulsive disorder patients. *Sci. Rep.* **6**, 31333; doi: 10.1038/srep31333 (2016).

## Supplementary Material

Supplementary Information

## Figures and Tables

**Table 1:** Demographic data for OCD patients and healthy control subjects.

	OCD patients	Controls	*P-*value
N	65	96	—
Sex, male/female	44/21	65/31	0.108
Age (yrs), mean (S.D.)	30.6 (5.1)	31.7 (7.6)	0.089
Education years, mean (S.D.)	11.5 (3.5)	10.7 (4.6)	0.091

**Table 2:** Number of probes found to be differentially methylated between OCD patients and healthy control subjects.

	Probes	Genes
All	8,417	2,190
Promoter associated	2,478	388
CpG islands	4,013	937
Both promoters associated and CpG islands	1,622	279

**Table 3:** Top 20 genes with high frequencies of methylation sites.

Gene	Frequency	Gene	Frequency
*BCYRN1*	57	*CLCN5*	23
*BCOR*	43	*AR*	23
*FGF13*	35	*ZNF75D*	23
*ARX*	31	*NHSL2*	21
*EFNB1*	26	*NHS*	21
*IKBKG*	26	*HLA-DRB1*	20
*TIMM17B*	25	*MID1IP1*	20
*PQBP1*	25	*DUSP9*	20
*G6PD*	24	*TSC22D3*	20
*ARHGAP6*	24	*LONRF3*	19

Abbreviation: brain cytoplasmic RNA 1 (*BCYRN1*), BCL-6 co-repressor (*BCOR*), fibroblast growth factor 13 (*FGF13*), aristaless related homeobox (*ARX*), ephrin B1 (*EFNB1*); inhibitor of kappa light polypeptide gene enhancer in B-cells, kinase gamma (*IKBKG*); translocase of inner mitochondrial membrane 17 homolog B (yeast) (*TIMM17B*); polyglutamine binding protein 1 (*PQBP1*); glucose-6-phosphate dehydrogenase (*G6PD*); Rho GTPase activating protein 6 (*ARHGAP6*); chloride voltage-gated channel 5 (*CLCN5*); androgen receptor (*AR*); (*ZNF75D*); (*NHSL2*); (*NHS*); major histocompatibility complex, class II, DR beta 1 (*HLA-DRB1*); MID1 interacting protein 1 (*MID1IP1*); dual specificity phosphatase 9 (*DUSP9*); TSC22 domain family member 3 (*TSC22D3*); LON peptidase N-terminal domain and ring finger 3 (*LONRF3*).

**Table 4:** Significant KEGG or GO pathways based on the genome wide methylation analysis.

KEGG or GO	Term	Count	Frequency (%)	*P*-Value	Pop Hits	Fold Enrichment	Bonferroni	Benjamini	FDR
hsa04810	Regulation of actin cytoskeleton	39	0.8872	3.19E-07	215	4.4262	5.67E-05	5.67E-05	3.92E-04
hsa04514	Cell adhesion molecules (CAMs)	28	0.7306	8.36E-06	132	4.2526	0.0014	7.43E-04	0.0102
hsa04144	Endocytosis	33	0.7306	2.27E-05	465	3.9255	0.0040	0.0013	0.0279
hsa04360	Axon guidance	23	2.0354	4.08E-05	125	1.9836	0.0072	0.0018	0.0501
hsa04540	Gap junction	17	1.4613	4.20E-05	89	2.3196	0.0074	0.0014	0.0516
hsa04730	Long-term depression	14	1.0438	1.23E-04	69	2.6350	0.0216	0.0036	0.1509
hsa04010	MAPK signaling pathway	36	0.7828	1.24E-04	267	3.2163	0.0218	0.0031	0.1526
hsa04720	Long-term potentiation	13	0.9394	1.55E-04	68	2.7723	0.0272	0.0034	0.1905
hsa04080	Neuroactive ligand-receptor interaction	32	1.7223	2.28E-04	256	1.9612	0.0397	0.0044	0.2796
GO:0003779	Actin binding	25	2.8409	6.52E-04	274	2.1454	0.4304	0.4304	1.0061
GO:0030528	Transcription regulator activity	87	0.6818	9.62E-04	1512	7.4254	0.5641	0.3398	1.4807
GO:0030695	GTPase regulator activity	31	3.5227	0.0021	404	1.8042	0.8472	0.4653	3.3185
GO:0016563	Transcription activator activity	27	9.8863	0.0028	410	1.3529	0.9132	0.4573	4.2975
GO:0022804	Active transmembrane transporter activity	24	3.5227	0.0029	363	1.7649	0.9247	0.4039	4.5410
GO:0022891	Substrate-specific transmembrane transporter activity	47	13.7500	0.0037	827	1.2673	0.9613	0.4185	5.6761
GO:0032395	MHC class II receptor activity	6	2.8409	0.0064	19	1.8032	0.9962	0.5491	9.5323
