# Supplementary material for: Genome-wide DNA methylation analysis in obsessive-compulsive disorder patients
Source: Sci Rep. 2016 Aug 16;6:31333. doi: 10.1038/srep31333 (PMC4985637; doi:10.1038/srep31333)

Genome-wide DNA methylation analysis in obsessive-compulsive disorder patients

WeihuaYue[1,2,#], Weiqiu Cheng[1,2,#], Zhaorui Liu[1,2], Yi Tang[1,2,3], Tianlan Lu[1,2], Dai Zhang[1,2,4], Muni Tang[5], & Yueqin Huang[1,2,*]

[1] Peking University Sixth Hospital & Institute of Mental Health, Beijing 100191, China.

[2] National Clinical Research Center for Mental Disorders & Key Laboratory of Mental Health, Ministry of Health (Peking University), Beijing 100191, China.

[3] Department of Mental Health, Guangdong Provincial People's Hospital, Guangzhou, China

[4] Peking-Tsinghua Center for Life Sciences / PKU-IDG/McGovern Institute for Brain Research, Peking University, Beijing 100871, China.

[5] Guangzhou Psychiatry Hospital, Guangzhou, 510080, China.

[#] These authors contributed to this manuscript equally.

[*]**Address correspondence to:** Prof. Yueqin Huang, M.D., Institute of Mental Health, Peking University, 51 Hua Yuan Bei Road, Beijing 100191, P. R. China. E-mail: dengy@mail.tsinghua.edu.cn or dryue@bjmu.edu.cn

Supplementary Figure 1. The principal component analysis (PCA) plots of the first five components of our sample. Plots of PC2-PC5 *v.s.* PC1, respectively, for methylation analysis of individuals with OCD included in the present study. Then we found that the genomic control factor (λ_{GC}) was about 1.094.

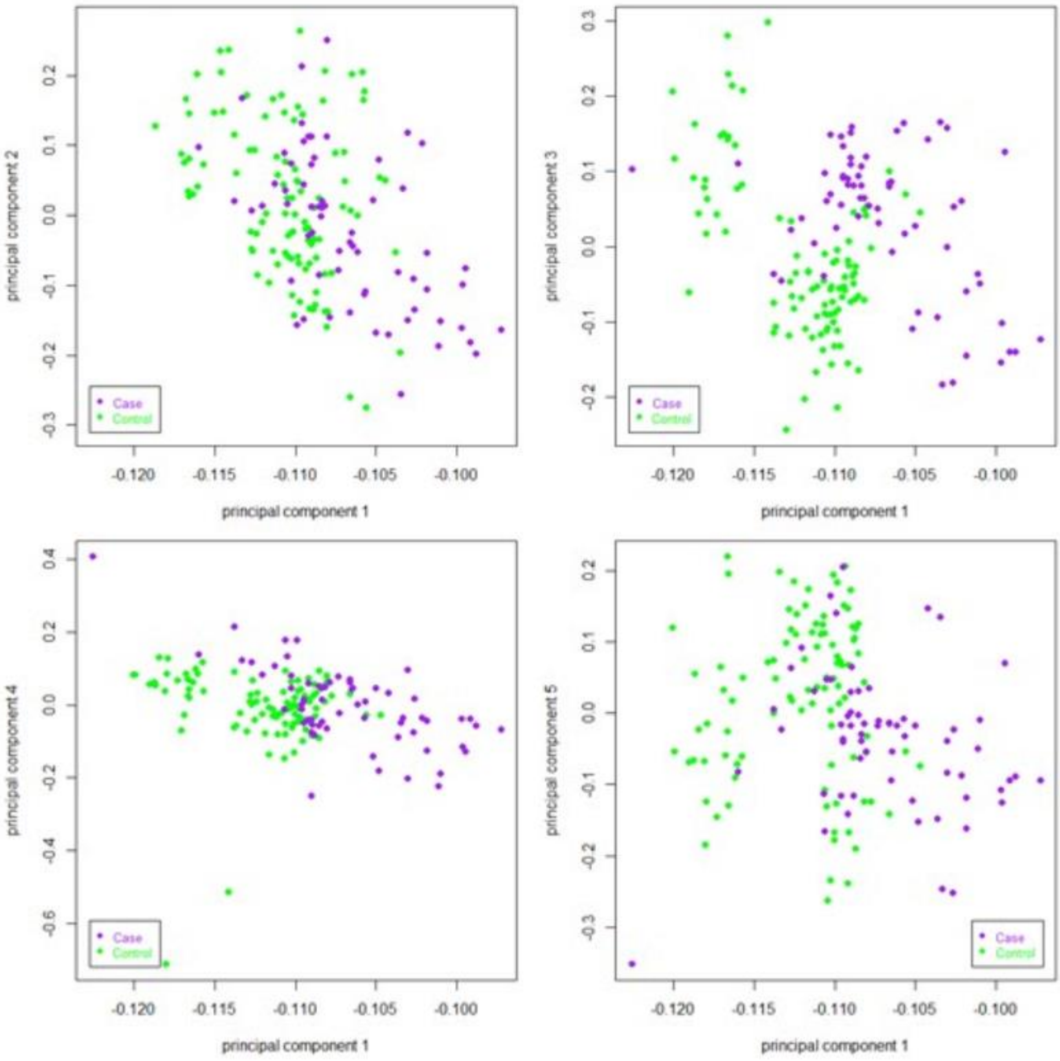

Supplementary Figure 2. Clustering profiles for selected methylation probes.

The clustering profiles for selected methylation probes (*diffscore* ≥ 20, delta β values > 0.1). Unsupervised clustering analysis of the top significant methylation probes revealed two distinct groups with significantly more people with OCD in cluster one compared with controls (67.74% cases *v.s.* 27.13% controls, $df = 1$, $P = 3.41E-07$). The abscissa axis represents id numbers of all the 65 OCD cases and 96 healthy control subjects and related clusters. The vertical axis represents clusters of the methylation probes. The color key represents Z-scores, from Green to Red as from -4 to 4.

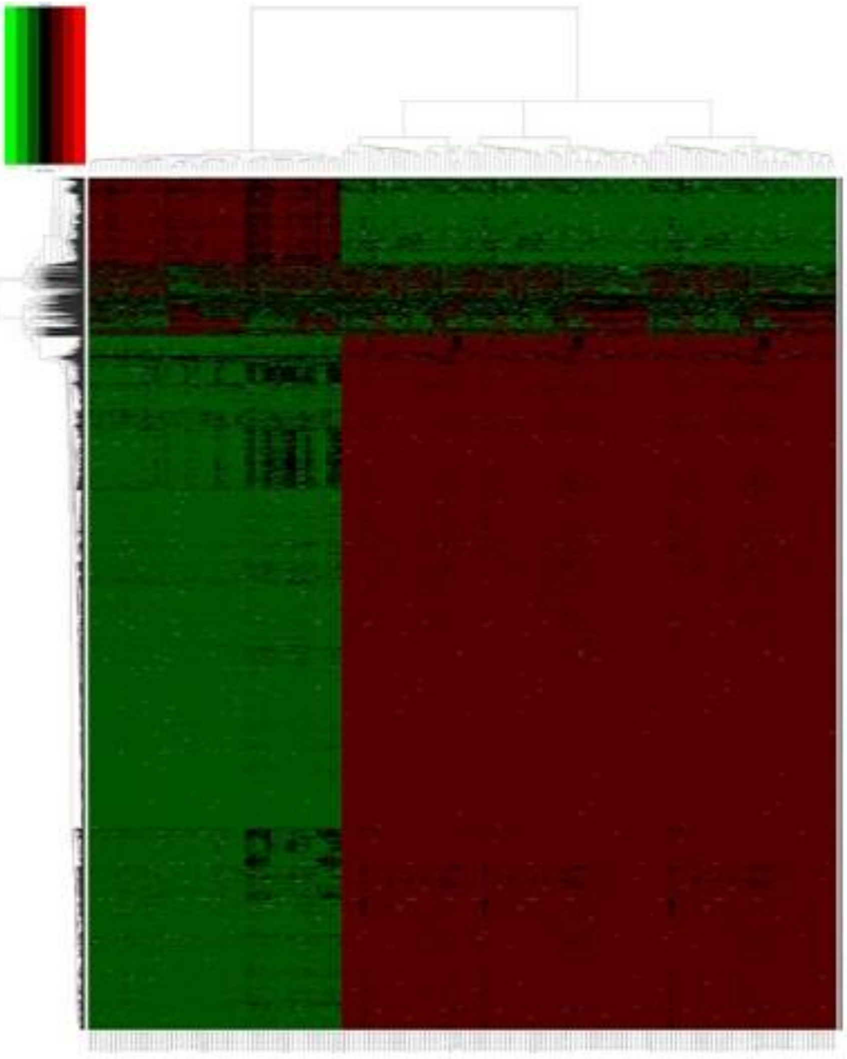

Supplement: Supplementary Information [file srep31333-s1.pdf]
